# Exploring the Evolution of Novel Enzyme Functions within Structurally Defined Protein Superfamilies

**DOI:** 10.1371/journal.pcbi.1002403

**Published:** 2012-03-01

**Authors:** Nicholas Furnham, Ian Sillitoe, Gemma L. Holliday, Alison L. Cuff, Roman A. Laskowski, Christine A. Orengo, Janet M. Thornton

**Affiliations:** 1 EMBL-EBI, Wellcome Trust Genome Campus, Hinxton, Cambridge, United Kingdom; 2 Institute of Structural and Molecular Biology, University College London, Darwin Building, London, United Kingdom; Bar Ilan University, Israel

## Abstract

In order to understand the evolution of enzyme reactions and to gain an overview of biological catalysis we have combined sequence and structural data to generate phylogenetic trees in an analysis of 276 structurally defined enzyme superfamilies, and used these to study how enzyme functions have evolved. We describe in detail the analysis of two superfamilies to illustrate different paradigms of enzyme evolution. Gathering together data from all the superfamilies supports and develops the observation that they have all evolved to act on a diverse set of substrates, whilst the evolution of new chemistry is much less common. Despite that, by bringing together so much data, we can provide a comprehensive overview of the most common and rare types of changes in function. Our analysis demonstrates on a larger scale than previously studied, that modifications in overall chemistry still occur, with all possible changes at the primary level of the Enzyme Commission (E.C.) classification observed to a greater or lesser extent. The phylogenetic trees map out the evolutionary route taken within a superfamily, as well as all the possible changes within a superfamily. This has been used to generate a matrix of observed exchanges from one enzyme function to another, revealing the scale and nature of enzyme evolution and that some types of exchanges between and within E.C. classes are more prevalent than others. Surprisingly a large proportion (71%) of all known enzyme functions are performed by this relatively small set of 276 superfamilies. This reinforces the hypothesis that relatively few ancient enzymatic domain superfamilies were progenitors for most of the chemistry required for life.

## Author Summary

Enzymes, as biological catalysts, are crucial to life. Understanding how enzymes have evolved to perform the wide variety of reactions found across all kingdoms of life is fundamental to a broad range of biological studies, especially those leading to new therapeutics. To unravel the evolution of novel enzyme function requires combining information on protein structure, sequence, phylogeny and chemistry (in terms of interacting small molecules and reaction mechanisms). We have developed a protocol for integrating this wide range of data, which we have applied to a relatively large number of families comprising some very diverse relatives. This has permitted us to present an initial overview of the evolution of novel enzyme functions, in which we observe that some changes in function between relatives are more common than others, with most of the functionality observed in nature confined to relatively few families. Moreover, we are able to identify the evolutionary route taken within a superfamily to change the enzyme function from one reaction to another. This information may help in predicting the function of an enzyme that has yet to be experimentally characterised as well as in designing new enzymes for industrial and medical purposes.

## Introduction

Enzymes, as biological catalysts, are critical for life, with a significant proportion (approximately 45%) of gene products annotated as having an enzyme function [1]. Moreover, they are often the targets for pharmaceutical drug development, with a large number of approved drugs acting to modify the behaviour of enzymes implicated in human disease as well as disease causing pathogens [2]. Much of our understanding about how enzymes perform their reaction chemistry is derived from the study of their three-dimensional atomic structure. In combination with a variety of chemical and biochemical experiments it is possible to propose reaction mechanisms for many different enzymes [3].

An enzyme's function, and in particular the reaction chemistry it catalyses, is encapsulated by a hierarchical classification system developed and maintained by the Enzyme Commission (E.C.) [4]. It consists of a four-level descriptor, with the first three levels broadly categorising the overall chemistry and the fourth level being a serial number that is assigned to differentiate the substrate specificity. It is important to note that there is no correlation between the differences between the reactions catalysed and the numerical identifiers in the E.C. classification; so E.C. number 1.1.1.1 is no more similar to 1.1.1.2 than it is to 1.1.1.25.

In general, it is possible to organise and classify proteins into families and superfamilies based on similarities between sequence and/or structure. Very distant relationships between proteins can usually be more successfully detected through analysis of their three-dimensional atomic structures rather than by sequence alone [5]. To this end, a number of classifications of protein three-dimensional structure have been developed to capture evolutionary relationships, most notably CATH [6] and SCOP [7]. Both of these classifications use protein structural domains as the discrete entity, with a protein being made up of one domain or more in which case it is described as having a multi-domain architecture (MDA). Domains often combine in multiple different ways creating different MDAs, often with different functions. Domains can be classified into superfamilies based on a detectable evolutionary relationship.

A number of studies have been undertaken on collections of superfamilies whose membership predominantly consists of enzyme structures and sequences [8], [9], [10], [11], [12], [13], [14] as well as numerous studies on single superfamilies, in addition to the insights made as part of enzyme design and re-design efforts [15], [16], [17], [18]. These analyses have observed that, whilst there is often conservation of some aspects of chemistry between relatives in enzyme superfamilies, there are examples of relatives which have diversified to perform very different functions (as defined by the overall reaction they perform), and/or to use different chemical mechanisms (the method by which the substrates transform), and/or act with different specificity. The route by which this functional diversity is achieved has proved to be complex. Changes to residues can subtly affect the binding of substrates, metal ions, or cofactors altering the chemistry performed. In some cases the recruitment or loss of domain partners can modulate the function [9]. All of these detailed studies have been undertaken manually on a relatively small number of superfamilies ranging in number from one to thirty. Understanding these evolutionary relationships is critical in the light of the continual flood of data from genomic projects, as it is often these insights that provide the best route for predicting function [19]. To address this challenge, we have developed FunTree [20], a system for exploring the functional relationships and their evolution between three dimensional structure and function in enzymatic superfamilies (http://www.ebi.ac.uk/thornton-srv/databases/FunTree/).

We apply the pipeline to analyse enzyme superfamilies in CATH, using robust structurally-informed multiple sequence alignments to build phylogenetic trees, which are then annotated with structural and functional data. Relationships between metabolites, obtained by exploiting tools for comparing small molecules, are displayed on the phylogenetic tree. We have chosen two specific superfamilies to illustrate the value of combining structural and functional data to explore evolutionary changes. Analyses of these functional changes in 276 well-defined enzyme superfamilies has allowed us to present a preliminary overview of the evolution of novel enzyme functions in order to begin to gather, catalogue and classify the emergence of the catalytic reactions necessary for life.

## Results

### Identifying and Grouping Related Domains

In order to understand the phylogenetic relationships and divergence of functions between protein domains, the first step is to identify related domains using both three dimensional data, based on CATH definitions, and sequence data. However, some of the structural superfamilies in CATH are highly diverse, containing very distant relatives with pairwise sequence similarities less than 10%. Whilst the core of the structural domain is generally well conserved, in some superfamilies distant relatives may exhibit different structural embellishments to this core [21], [22]. In these cases it can be very difficult to align all the structural domain representatives of the superfamily robustly.

Therefore, we have developed a protocol for identifying groups of structurally similar relatives within a superfamily that can be well aligned and superimposed in 3D. These are termed ‘structurally similar groups’ (SSG). Sequence relatives were added to each SSG and then all sequences were multiply aligned and used to derive the phylogenetic tree for that SSG (see Materials and Methods for full details). An established species tree [23] guided the phylogenetic tree and bootstrap values at the braches are shown. Modification of a domain's function can also be achieved by changing the multi-domain context i.e. by changing the domain partners [24] or through the duplication and diversification of domains [25]. To explore how the addition of domains can affect a given domain's function, a different sub-clustering of the superfamily was made based on the unique multi-domain architecture identified using ArchSchema [26]. Proteins that contain the superfamily domains in the same order are clustered together. These clusters are termed multi-domain architecture (MDA) groups.

We used MACiE [27], a manually curated database of enzyme mechanisms designed to provide a wide range of E.C. defined functions, to identify 276 enzymatic CATH superfamilies with adequate structural and functional data suitable for processing by the FunTree pipeline. The superfamilies included representatives from 189 different fold groups and all four CATH classes.

**Figure 1:**
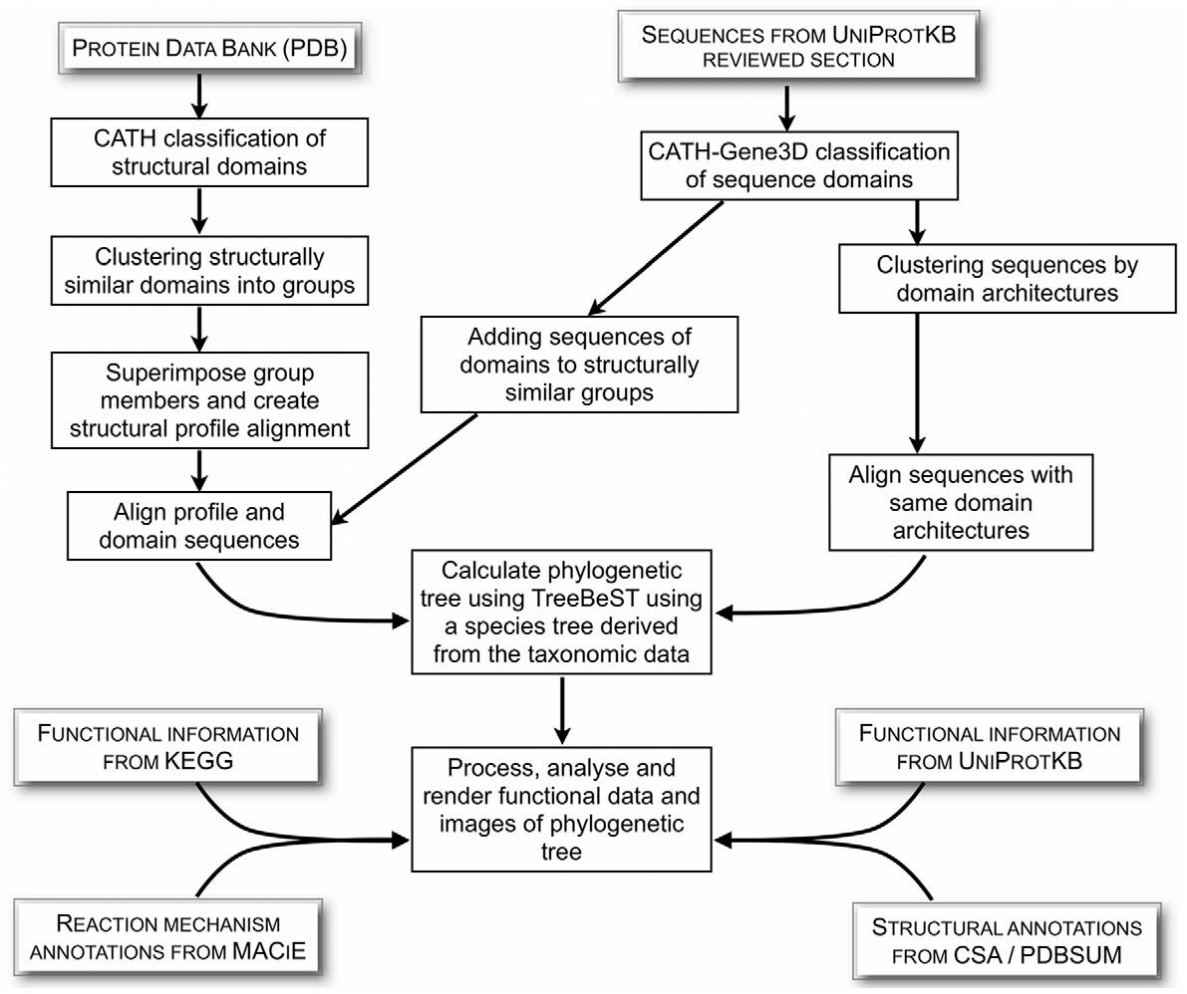
An Overview of the Pipeline for Generating Structurally Similar Groups, Multi-Domain Architecture Groups and their Respective Phylogenetic Trees.

For each superfamily and their SSG/MDA clusters, we generated a number of visualisations of the data. The principal visualisation is the sequence and structure based phylogenetic tree decorated with its associated annotations. The results of the small molecule clustering are rendered as a separate dendrogram and an overview of the functional variability is supplied as an un-rooted tree of the E.C. hierarchy. Additionally, at the superfamily level, we show the multi-domain architectures using the ArchSchema graphing software. A summary of the protocol is shown in Figure 1.

### Analysis of Two Superfamilies

FunTree intimately links the chemical functions (as defined by the reactions and the substrates catalysed) of a superfamily of enzymes with their structures and evolutionary history. We use two superfamilies to illustrate how FunTree captures and describes changes in function. These two superfamilies exemplify two different paradigms of enzyme evolution. We then integrate the functional changes of all 276 superfamilies, giving for the first time an overview of our current knowledge of the scope and evolution of the ‘reactions of life’ as known today.

### Phosphatidylinositol-Phosphodiesterase Superfamily

**Figure 2:**
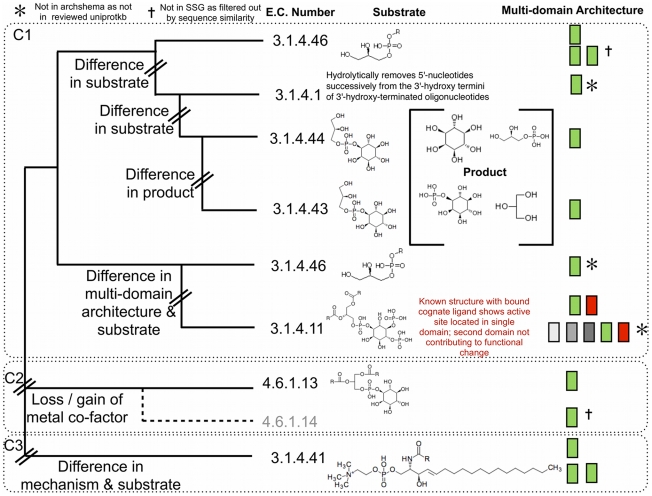
Summary of Phylogenetic, Functional, Metabolite and Domain Architectures for the Phosphatidylinositol-phosphodiesterase Superfamily. A diagrammatic representation of the FunTree phylogenetic tree with associated functional data and multi-domain architectures from ArchSchema. Each domain is given a unique colour, with the domain of focus coloured green. Three major clades (C1–C3) are highlighted. Within the first group a number of functional sub-groups can be observed, with differences in function defined by changes in substrate or product formed. The presence of additional domains does not change function.

**Figure 3:**
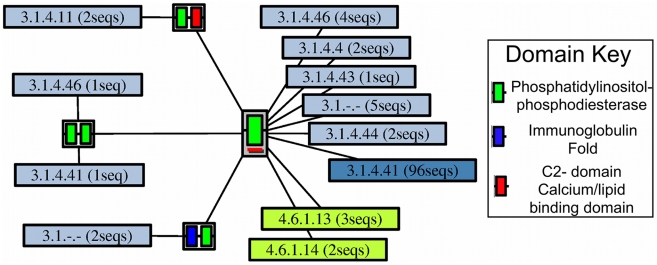
Multi-Domain Architectures Defined by Phosphatidylinositol-Phosphodiesterase Domain. An ArchSchema graph showing the multiple domain architectures found in the phosphatidylinositol-phosphodiesterase superfamily. Each node represents a unique multiple domain architecture, with a red line under the node indicating that a structure exists. Also shown are the E.C. numbers (and the number of sequences which have them in brackets) found for each MDA. Each E.C. class is coloured separately, with the intensity of the colour proportional to the number of sequences that have that E.C. assigned. Only domain architectures for sequences that are annotated in the reviewed section of UniProtKB are shown.

The phosphatidylinositol (PI) phosphodiesterase superfamily (CATH id 3.20.20.190) is relatively structurally conserved with all domain structure representatives in one structurally similar group (SSG). There are only four different MDAs, with only one change in domain partner having a corresponding change in function (see Figures 2 and 3 and Figure S1). However, detailed structural analysis of the binding of the cognate ligand for this MDA reveals that the second domain does not have any direct molecular functional role (see Figure S2). Thus the single domain performs all the molecular functionality observed within this superfamily.

The phylogenetic tree for this SSG has three distinct clades. The first clade (C1) contains a variety of general and specific phosphodiesterases (see Text S1) from bacteria and eukaryotes all performing a hydrolase reaction using a metal co-factor and the same catalytic machinery, although relatives use different substrates and generate different products.

In the second clade (C2) in the phylogenetic tree the enzymes become lyases, which are found in bacteria and protozoan trypanosomes rather than mammals, changing between a hydrolase (E.C. 3.1.4.11) and a lyase (E.C. 4.6.1.13/4.6.1.14). The mammalian and bacterial phosphodiesterases all follow the same initial mechanism, the intramolecular addition of a hydroxyl group adjacent to the phosphate with elimination of the first alcohol substrate; however, for the bacterial enzymes in the second clade the metal cofactor is not present. This results in the cyclic intermediate leaving the active site prior to hydrolysis (thus defining a lyase rather than a hydrolase) whereas in the mammalian case, the intermediate is strongly bound and hydrolysis occurs within the enzyme. In both cases a pair of histidine residues act as general acid/base catalysts in the mechanism. The structure-informed sequence alignment reveals that none of the three metal chelating residues are conserved between these two clades.

A fourth phosphodiesterase enzyme is also found in the third clade (C3) but this acts on 2-lysophosphatidylcholine (E.C. 3.1.4.41) and is involved in the generation of venom in Sicariid spiders [28]. It utilizes a very different substrate to the rest of the superfamily and is reported to have a markedly different mechanism [29]. Although the two histidine residues and the metal cofactor are still present, the histidines act in a nucleophilic manner forming a covalent bond between the phosphate and enzyme (see Figure S3). Other residues are less conserved (see Figure S4). Taken together, the data suggest that the changes in mechanism have occurred through modulation of existing residues rather than gain/loss of structural elements or loop regions. The outlying position of the clade in the phylogenetic tree, in combination with the available supporting literature catalogued by MACiE, clearly supports a mechanistic change for this grouping. It is not possible to determine the cause of this change in mechanism with currently available information.

Analysis of changes in E.C. class, sub-class (2^nd^ level) and substrate specificity (4^th^ level) within a superfamily indicate transitions that have occurred since the protein diverged from its common ancestor. This superfamily has undergone a single transition between the hydrolase and lyase classes with no changes occurring at the sub-class level (summary shown in Figure S5A), and lyases are only seen in bacteria and trypanosome protozoa. There has been a diversification in the substrates within the hydrolases, which are known to utilize five different substrates to date. No such diversification in substrate utilization is seen for the lyase performing enzymes within this superfamily.

This superfamily provides an interesting example of relatives undertaking similar overall functions but with quite different mechanisms despite similarity in structures and identical catalytic residues in the same locations. It also illustrates a complex enzyme evolution that would have been difficult to predict from a simple examination of structures and active sites but has been revealed by bringing together a diverse range of information in FunTree.

### Ntn Type Amide Hydrolasing Superfamily

**Figure 4:**
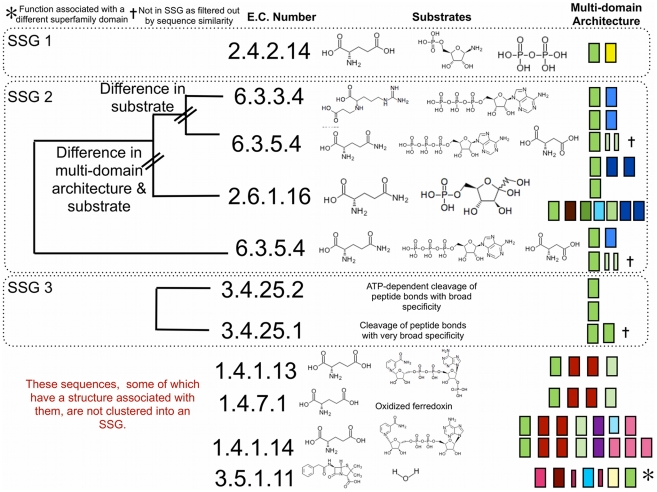
Summary of Phylogenetic, Functional, Metabolite and Domain Architectures for Ntn-type Amide Hydrolase Superfamily. The superfamily is divided into three structurally similar groups, with a diagrammatic version of the FunTree phylogenetic tree shown for each, as well as functional, substrate and multi-domain architecture data. Each domain is given a unique colour, with the domain of focus coloured green.

The Ntn-terminal type amide hydrolasing superfamily (CATH id 3.60.20.10) is relatively structurally diverse, with three SSGs (See Figure 4 and Figure S6). SSG 1 contains just the amidophosphoribosyltransferase (E.C. 2.4.2.14). SSG 2 contains the glutamine-dependent asparagine synthetases (E.C. 6.3.5.4) and the arginine beta-lactam-synthase (E.C. 6.3.3.4) as well as glutamine-fructose-6-phosphate transaminases (E.C. 2.6.1.16). All use different substrates, though some are shared (glutamate between E.C. 2.6.1.16 and E.C. 6.3.5.4 and ATP between E.C. 6.3.5.4 and E.C. 6.3.3.4) and have different MDAs.

Some of the domain relatives in this superfamily form part of the proteasome, a large and important multi-subunit complex found in all major kingdoms that undertakes the vital function of eliminating proteins that are mis-folded, harmful or unnecessary [30]. In this context, the enzyme loses its preference for glutamine and acts generally to cleave the peptide bond (E.C. 3.4.25.1/E.C. 3.4.25.2), though the preference for glutamine has been observed with post-glutamyl peptidolytic activity using synthetic peptides [31]. All of the proteasome related structures and sequences are found in SSG 3 and are grouped with a proteasome-related protein-degradation machine HsIVU [32]. All consist of members with a single domain, though *in vivo* they are part of a large multi-subunit machine.

A separate set of structures and sequences, which are singleton SSGs (ie they contain only one structure in the SSG), are associated with glutamate synthesis (E.C. 1.4.1.13, 1.4.1.14 and 1.4.7.1). All use glutamate but differ in the co-factor that they use which varies between NAD+, NADP+ and ferredoxin. There is another singleton with a very different function (E.C. 3.5.1.11 - penicillin amidase) associated with it. Examination of the known structures and mechanisms reveals that this function is performed by one of the other domains in the enzyme and is also found in some multi-domain enzymes where the Ntn-terminal type amide hydrolasing domain is not found. This highlights the care needed when associating function with an enzyme with multiple domains.

**Figure 5:**
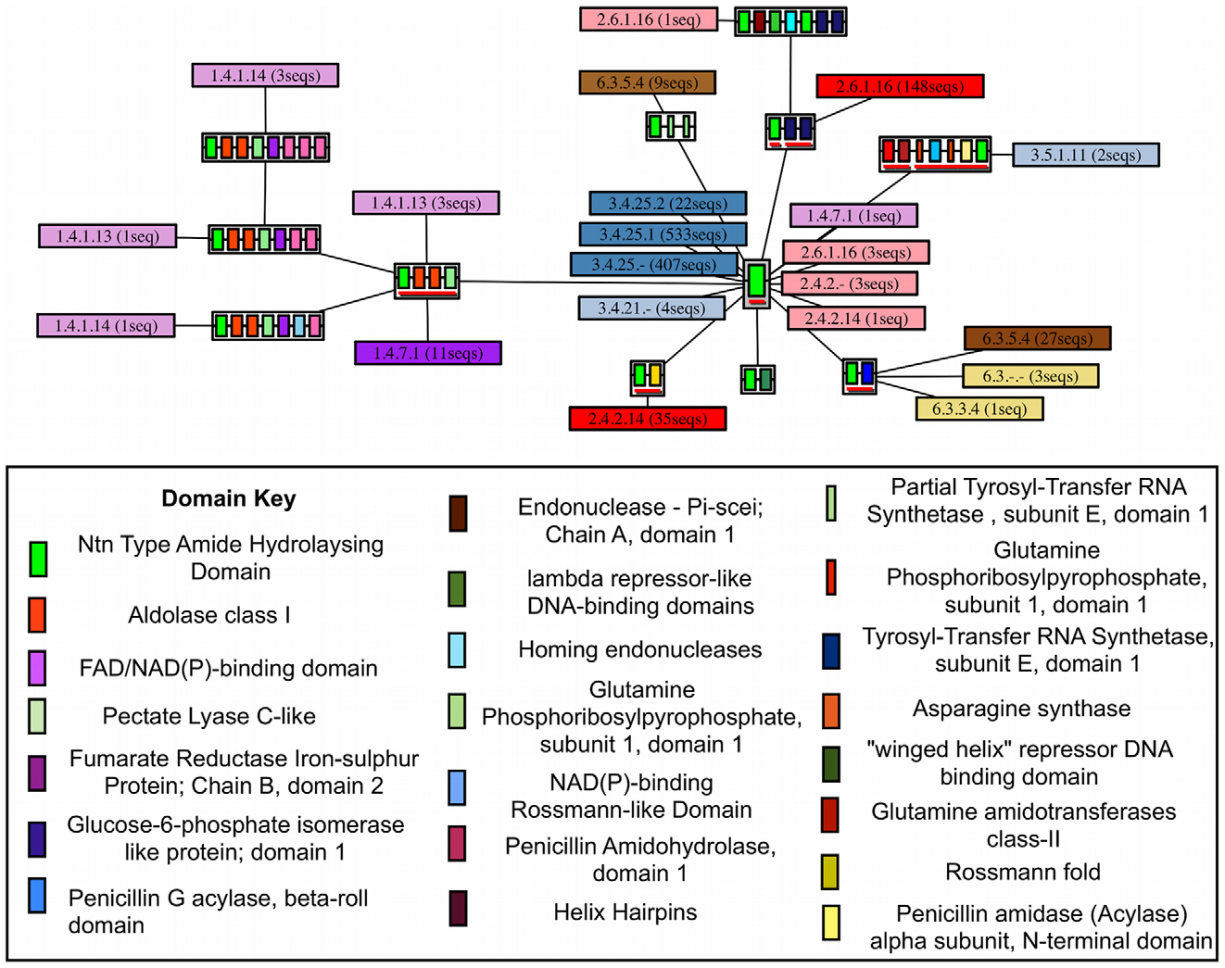
Multi-Domain Architectures as Defined by Ntn Type Amide Hydrolasing Domain. The ArchSchema graph showing MDAs as defined by the Ntn type amide hydrolasing domain. It should be noted that the MDA representing the single domain has cataloged two sequences that have the amidophosphoribosyltransferase function. These sequences represent two truncated sequences (the truncations resulting from a frame-shift), with the full sequence comprising two domains (highlighted by a caution remark in the UniProtKB record). The two truncated sequences inherit the function from the full sequence. Likewise a glutamate synthase function is also ascribed to a sequence in this MDA but comes from a sequence fragment and is likely to be a longer sequence with more domains. Though rare, this highlights the care that needs to be taken when analysing sequence annotations.

We performed the same analysis as for the previous superfamily cataloguing changes in E.C. numbers at various levels (see Figure S5B). This is a more complicated superfamily, with transitions between four classes (oxidoreductases, transferases, hydrolases and ligases). There are also changes within the transferase class at the sub-class level, indicating a change to the group that is being transferred. In addition there is diversification in substrate specificity within the oxidoreductases and hydrolases. The domains in these SSGs and the unclustered singletons are present in different domain combinations (see Figure 4). It can be seen from the ArchSchema graph (see Figure 5) that changes in function correlate with changes in MDA (i.e. domains with the same MDA have the same function). In fact structural differences between the domains are largely related to unstructured linker regions that are in close proximity to the domain partners and may be facilitating interactions with the domain partners. In SSG3 a helical embellishment to the domain core is involved in mediating contacts with protein partners in the biological unit. The correlation observed between MDAs and E.C. functions suggests that changes in the domain partners contribute to changes in the function. Analysis of mechanistic and structural domain partners (details provided in Figures S7 and S8 and Text S1) reveals that the Ntn-terminal type amide hydrolasing domain primarily generates an amine (generally ammonia) from hydrolytic cleavage of the amide bond, which is then used by a second domain in a variety of ways. It is the combinations of domains that produce the range of functions observed within this superfamily.

### Analysis of Superfamilies, Structurally Similar Groups and Domain Architectures

**Figure 6:**
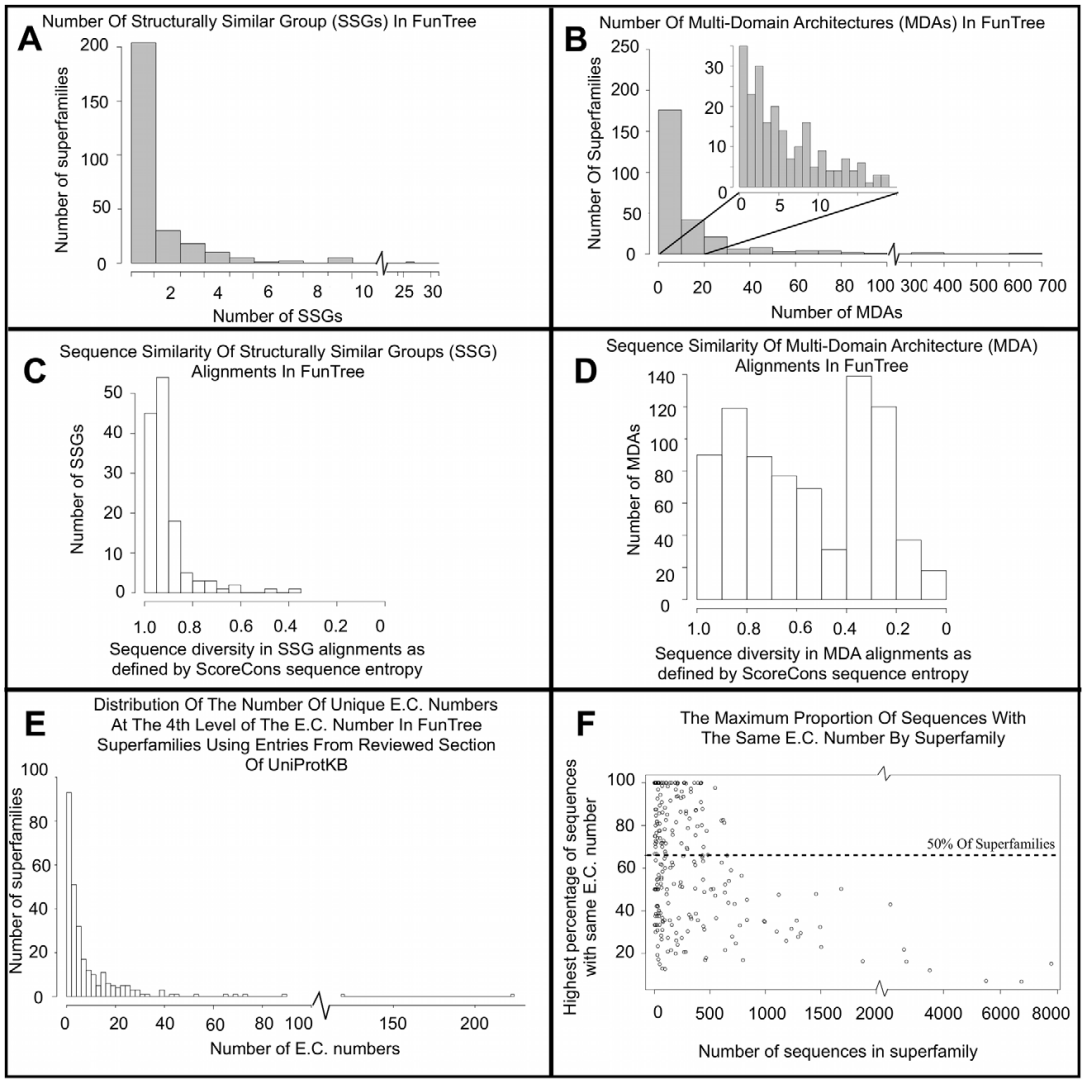
Structural and functional diversity of the 276 superfamilies. **A & B.** Distribution of the number of structurally similar groups and unique multi-domain architectures in these superfamilies. **C.** Distribution of sequence conservation in the alignments for structurally similar groups (SSG) and **D.** multi-domain architectures (MDA), as measured by ScoreCons. Although some MDAs are quite diverse, others appear quite conserved, which may be due to some MDAs having relatively few sequences associated with them. **E.** The distribution of the number of fully described (to the fourth level) E.C. numbers across all 276 superfamilies. **F.** Shows the largest percentage of sequences with the same E.C. number compared to size of the superfamily observed by the number of sequences with a fully classified E.C. number in the superfamily. A dashed line shows that 50% of superfamilies have greater than 65% of their sequences with the same E.C. number.

The 276 superfamilies processed in the current version of FunTree account for approximately 15% of all known domain assignments in CATH-Gene3D. The top 10% of superfamilies in our data (<30 superfamilies) ranked by number of SSGs account for 1,064,627 sequences (49% of sequences in our data) with on average 5 SSGs per superfamily. The rest of the superfamilies have on average only one SSG per superfamily. Whilst the majority (∼75%) of the superfamilies contain only one structurally similar group (SSG), indicating that most superfamilies show limited structural divergence (see Figure 6A) a few superfamilies have a very large number of SSGs with the largest being the P-loop containing nucleotide triphosphate hydrolases with 27 SSGs. Although the structures show limited divergence, if the sequence diversity is measured using ScoreCons [33], the majority of SSGs are highly diverse (Figure 6C). Most superfamilies contain fewer than ten different multi-domain architectures (Figure 6B) and compared to the SSG alignments, the degree of sequence diversity within MDAs is relatively evenly spread with some being highly diverse and others very conserved (Figure 6D). This accords with previous observations [34].

There is some correlation between the number of SSGs and MDAs (Pearson correlation value of 0.77). This is expected since structural modifications and decorations to the central core facilitate new interactions with domain partners, as in the Ntn-terminal type amide hydrolasing superfamily. However, the number of unique multi-domain architectures in each superfamily correlates poorly with the number of unique E.C. numbers (Pearson correlation value of 0.57). This indicates that, although in some families a domain partner brings an increase in functional diversity, surprisingly there are a number of families where most of the functionality is present in the single domain, for example the terpene synthases/cyclases and the phosphatidylinositol-phosphodiesterases.

The distribution of the number of associated functions for each superfamily, as defined by the E.C. number and our general observations indicate that, although the majority of members of an enzymatic superfamily share a common function, some superfamilies have the ability to accommodate many diverse functions (see Figures 6E and 6F). The top five ‘polymath’ enzyme superfamilies with multiple functions are: the NADH binding domain, P-loop containing nucleotide triphosphate hydrolases, Class 1 aldolases, S-adenosyl-L-methionine-dependent methyltransferases, trypsin-like serine proteases, and Type I PLP-dependent aspartate aminotransferase-like superfamily. All these have more than 50 functions as defined by E.C. to the 4^th^ level in each superfamily. Although a significant number of our superfamilies have both general chemistry and substrate diversity present in the superfamily e.g. the NADH binding domain superfamily, most functional diversity comes from utilising multiple substrates with 177 (67%) superfamilies where changes in E.C. occur only at level 4 i.e. change in substrate specificity. This builds upon our previous smaller scale studies [35] as well as observations made by Glasner and co-workers [12] and more recently by Khersonsky and co-workers [36].

E.C. numbers attributed to these 276 superfamilies (including relatives where the domain is in different MDA contexts) account for 71% of the 2,676 E.C. numbers assigned to known enzymes, with the E.C. numbers associated with single domain enzymes accounting for approximately 36%. The high coverage of enzyme functionality from just 276 superfamilies, given that this represents only 15% of known domains, is surprising. Moreover, just 45 superfamilies account for 50%, of all sequences assigned E.C. numbers with 31 superfamilies in which the single domain accounts for 25%.

From this we can postulate that a limited repertoire of structural frameworks has evolved to carry out a large proportion of reactions required for all of life. Moreover, it is clear that generating new chemistry does not necessarily require large leaps, such as the evolution of novel protein structures or large structural re-arrangements, but can be made by small local changes e.g. residue substitutions or small insertions or deletions. Functional changes can also arise from changes in MDA and less frequently insertion/deletion of unstructured regions. This is perhaps not surprising since residue changes in the active site can easily induce changes in chemistry. Superfamilies supporting a wide range of enzyme functions predominantly adopt one of a few relatively highly populated superfamilies, such as the TIM barrel or Rossmann-like fold, which both possess large surface clefts likely to tolerate residue mutations [21].

We also observe that the addition of another domain or set of domains can bring a function associated solely with those domains and not with the superfamily domain (see Figure S9 and S10) i.e. acquisition of function by domain addition. These domains can bring confusion as to where the function is originating and the role (if any) that the superfamily domain under scrutiny contributes to that function. The contribution of these additional domains to the functional repertoire of a superfamily has been taken into account.

### The E.C. Exchange Matrix

**Figure 7:**
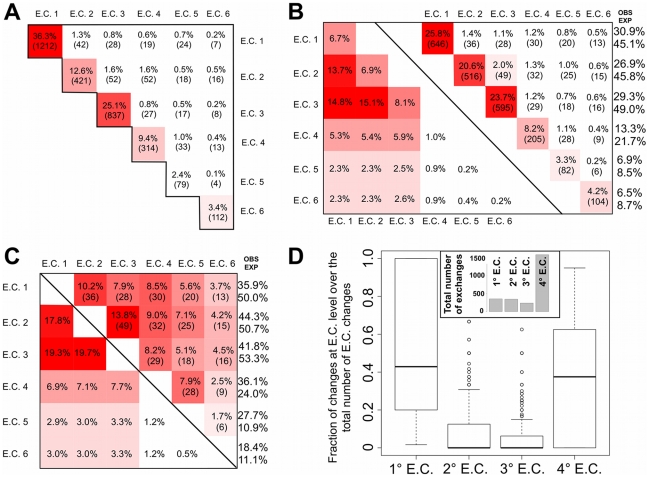
Changes in Function within 276 Superfamilies. **A:** A heatmap showing the cumulative changes in all superfamilies where a change is observed based on the differences in E.C. annotations at the class level in a superfamily and uses the phylogenetic tree to infer the order in which changes have occurred. These counts do not take into account changes that occur between SSGs and so need to be viewed in conjunction with the counts in B (right of the diagonal). The colour intensity indicates the number of times a change in E.C. class occurs. The matrix shows the percentage of changes (with total counts in brackets) in E.C. class observed across all 276 superfamilies. Along the matrix diagonal the number of changes occurring within the E.C. class to the 4^th^ level of the E.C. number. **B.** A similar heatmap to that described for A, but using all the possible combinations of E.C. found in a superfamily. The top right of the matrix shows the observed percentage of changes, with actual count totals in brackets, while the lower right half shows the percentage of changes expected based on a random simulation of E.C. changes. To the right of the matrix the observed (OBS) and expected (EXP) percentage of changes for each E.C. class are shown. **C.** The same exchanges as described for B but concentrating on the interchanges between classes. **D.** A box plot showing the proportion of E.C. changes in a superfamily by E.C. level (i.e. derived from data in A top right of matrix). For example if a superfamily has 2 changes at E.C. level 1 and 3 changes at level 4, then the primary E.C. level has contributed 2/5 and the fourth level has contributed 3/5. These fractions are catalogued across all superfamilies in the plot. The insert shows the total number of observed exchanges at each class level. All interchanges shown in A to D exclude those that are being contributed by ‘confusion domains’ detailed in Figure S12.

The major reason for this work was to explore the evolution of enzyme function; therefore we examined the range of E.C. classes found within each superfamily. We did this first by using the phylogenetic tree derived by FunTree to identify the evolutionary route taken within a superfamily to exchange the enzyme function from one reaction to another. It can be seen from Figure 7A that overall the exchanges within an E.C. class are proportionally the most abundant, while exchanges between classes are generally fewer.

Using the evolutionary route we are able to determine the structural changes associated with the functional shifts, which can include modulation of functional residues or through the loss or gain of loop regions. For all changes in function in the phylogenetic tree found at a bifurcation with a single function represented on each side of the divide, we catalogued whether known catalytic site residues were aligned to gapped (with a minimum gap space of three places) region. The requirement for high quality annotations and single functional changes means that the total number of exchanges catalogued is small (1,107 compared to 3,335 exchanges catalogued in all trees). Only 5% of functional shifts are associated with the addition/deletion of loops and these functional changes split equally between changes in E.C. classes and within E.C. classes.

In addition, we also catalogued where these exchanges corresponded to a change in the multi-domain architecture (MDA). This showed that 27% of changes were associated with changes in the multi-domain architecture, although this is an upper estimate as it includes enzymes where acquisition of function is through the addition of a domain and not by changes in the domain under scrutiny.

However, by counting E.C. exchanges using the FunTree derived phylogenetic trees E.C. exchanges occurring between SSGs will be missed (see Figure S11A). In addition, some EC changes occurring more than once during evolution will be double counted. Whilst, this information may provide interesting insights as to which changes in chemistry have been more favoured during evolution, we were interested in understanding the full range of possible E.C. changes within a superfamily. Therefore, we have also explored E.C. exchanges by counting all possible changes within a superfamily. For example, if one member of a superfamily is a transferase (E.C. 2) and another a hydrolase (E.C. 3), it is reasonable to assume that a direct transition between one class and another may have occurred at some point since the proteins diverged from a common ancestor. Therefore, all-by all counting (see Figure 7B) allows the possibility of changes within superfamilies that are not captured by known sequence relatives or that have been missed due to the necessity of building separate trees for different structural clusters in the superfamily. For example, for the exchanges occurring within the E.C. 1 class using the phylogenetic tree to count all exchanges misses 85 possible exchanges.

By combining information from the 276 superfamilies we can generate an E.C. exchange matrix summing all possible changes within the superfamilies. This gives 2502 unique exchanges at all levels of the E.C. classification, with 354 exchanges (approximately 15% of total exchanges observed) in the primary E.C. level.

In addition to counting all possible changes, the all-by-all counting permitted a random model of expected changes to be generated based on all the E.C. numbers present in the dataset, with pairs of E.C. numbers picked at random to generate the matrix. Comparison using a χ^2^ test of the two matrices shows they are significantly (P-value <10^−16^) different. The most striking difference is that exchanges within a class (along the matrix diagonal), are much more common than would be expected. These interchanges represent 85% of all changes observed, with most occurring in the first three classes as expected due to the higher number of divisions in E.C. 1, E.C. 2 and E.C. 3.

If the exchanges **within** a class are removed from the matrices (see Figure 7C) we observe that, as expected, the number of divisions in the classification of each class heavily dominates the exchanges calculated in the random model. However, this distribution is not seen in the observed exchanges, with exchanges between the oxidoreductase, transferase, hydrolase classes occurring less than expected, while exchanges between the lyase, isomerase and ligase classes occurring more frequently than expected. In some cases the addition or removal of a step in the reaction changes the enzymes classification from one class to another, as exemplified in the phosphatidylinositol phosphodiesterase superfamily, exchanging from hydrolase to lyase. Some of the changes between classes reflect the structure of the E.C. classification. For example, there are some instances where an enzyme is classified as a lyase but includes hydrolysis as part of its mechanism.

If the changes are considered for just the single domain enzymes (see Figures S11B and S11C), though the absolute counts are less by approximately 68%, they are proportionally similar to those found across the whole superfamily. This reinforces the observations made earlier that changes in chemistry and specificity are often achieved within a single domain alone.

Figure 7D, based on the exchanges reported in Figure 7B, shows the proportion in each superfamily of exchanges occurring at each level of the E.C. classification across all 276 superfamilies. Superfamilies show a variety of behaviours, with some only changing at the fourth level, whilst others (and often smaller families) are dominated by changes at the primary level. As the second or third level broadly represent the bond type or functional group, the lack of observed changes at this level indicates that changes within a superfamily to the bond type/functional group are much less likely than changes to overall chemistry or substrate specificity. The differences in the number of changes in the 1^st^ level and 4^th^ level of the E.C. number over the 2^nd^ and 3^rd^ levels is interesting, but difficult to interpret without detailed analysis of the chemistry of the reaction. This work is in progress. It clearly reflects the structure of the E.C. classification and needs deeper analysis. The overall distribution of total changes at each level of the E.C. classification (see the insert to Figure 7D) shows that changes at each of the first three levels are less likely than a change at the fourth level. Also, over all superfamilies, changes in the chemistry (the combination of the first three E.C. levels) are less likely than changes in substrate specificity (the fourth level).

## Discussion

In this study we have benefited from exploring distant evolutionary relationships, captured by structural comparisons in the CATH classification. Whilst some extremely distant relatives cannot easily be aligned because of the degree of structural change during evolution, our analyses have exploited robust structural groupings within CATH superfamilies to identify general trends in the evolution of function in enzyme superfamilies. A caveat to our study relates to the problems in functional annotations in public databases, some of which are unreliable and some of which can be limited, for example by the lack of promiscuity data, which is rarely adequately explored. In addition, the E.C. classification system does not lend itself easily to providing an automatic means to quantitatively compare two reactions, since it does not capture the mechanism of the enzyme.

A significant proportion of the reactions required for life are performed by a relatively small number of superfamilies so it can be postulated that a few ancient enzymatic domain superfamilies were progenitors for most of the chemistry required for life, this considerably develops previous observations [37]. Using the phylogenetic trees to define the evolutionary route taken within a superfamily to change function, we were able to generate the E.C. change matrix. The large numbers of changes at the E.C. 4^th^ level in the summary of E.C. changes in phylogentic trees compared to the low number of E.C. class changes indicates that changes in specificity occur mostly at the leaves of the trees, while more fundamental changes in chemistry occur at the root of the tree. Further work is required to ascertain when in evolution these changes occurred. Therefore a large amount of enzyme diversity occurs through evolution rather than de novo invention. Although, of course, new enzymes must have evolved at some stage, probably very early in the evolution of life. To identify the small number of ‘original’ enzyme progenitors requires more work and more experimental data.

This study focuses on divergent evolution and does not consider cases of parallel evolution of enzyme function where two completely unrelated enzymes are able to catalyse the same reaction, sometimes by different mechanisms. Current analysis has shown that on average there are about two unrelated enzymes for each E.C number [38]. Previous studies have suggested some evidence for convergent evolution, and this needs further exploration.

We found diversity of function within superfamilies at all levels of the enzyme classification, with changes between some E.C. classes occurring more frequently than others, though this in part reflects the human-devised nomenclature. There is also a large variation between individual superfamilies and SSGs/MDAs; some are highly diverse while others are almost mono-functional. Most seem to possess diversity at the 3rd level of E.C. or above, indicating a change in reaction chemistry as well as possessing diversity in the substrate metabolites. This can be driven by plasticity of the active site as well as the ability to recruit domain partners (e.g. Ntn-type amide hydrolases superfamily).

Our analysis has reinforced the observation that enzyme evolution is incredibly complex, with many different routes being taken to obtain different reactions, mechanisms and specificities within a superfamily. Such routes involve gene duplication followed by sub-functionalisation. The basis of such sub- functionalisation can be twofold: Firstly, by alteration of the enzyme structure, either by mutations, local insertions and/or deletions within a domain, or secondly by changes in multi-domain architecture as exemplified in the Ntn-terminal type amide hydrolasing superfamily. From a chemistry perspective, these structural changes can affect the overall reaction or the substrates, as exemplified in the phosphatidylinositol phosphodiesterase superfamily.

The tools we have developed in FunTree bring together all the relevant data to help understand the molecular basis for each reaction change, but still require detailed inspection of enzyme mechanisms (as captured in MACiE) and three-dimensional structures to achieve a thorough understanding much has we have already done for the phosphatidylinositol phosphodiesterase and Ntn-type amide hydrolases superfamilies. These superfamilies provide exemplars of the type of analysis that is possible using the resource. The primary level of the E.C. classification can be summarised by simple chemical reactions (see Figure S12). We hoped it might be possible to understand the E.C. exchange matrix based on the simple reactions. However these overall reactions have many steps, with typically three to six steps and varying between one and sixteen steps. To understand and extract the paradigms that underlie, for example, a change from a lyase to transferase, we need to inspect all lyase-transferase exchanges to see if common routes exist. We can then ask what are the most common paradigms? Knowing a reaction, can we predict which exchanges are most likely to occur? Can we predict new substrates or new chemistries? By beginning to gather, catalogue and classify the emergence of catalytic reactions we can analyse such shifts in functionality across and within enzyme superfamilies and this may help in designing new enzymes as well as aid in function prediction from sequence and structure.

## Materials and Methods

FunTree is based on domain superfamilies defined by the CATH ‘H’ level. The superfamilies used in this study were selected based on possessing active site residues, as identified from the MACiE database, located on a single CATH domain. These superfamilies were subsequently grouped according to structural similarity and according to their domain partners.

### Generating Structurally Similar Groups (SSGs) and their Sequence Alignments

Although domains in a superfamily share a common structural core, distant homologues can show considerable variation outside this core, making it hard to robustly superimpose all domains within some superfamilies. Therefore we identified structurally similar groups (SSGs) of non-redundant domains with greater than 35% sequence identity which could be superimposed with a root mean squared deviation of less than 9 Å. Multiple structure alignments were generated using CORA [39]. These alignments were used to generate a structure based sequence profile for the SSG using MELODY (part of the FUGUE [40] fold recognition software). Sequence relatives for each CATH superfamily are provided by CATH-Gene3D and included only if part of the reviewed section of UniProtKB [41]. These are scanned against sequences of all the CATH superfamily domains of known structure using BLASTp [42] to determine which SSG they should be assigned to. They are then aligned to the structure-based sequenced profile of that SSG using FUGUEALI (also part of the FUGUE software). These structure-based sequence alignments are used to perform the phylogenetic analysis.

### Generating Multi-Domain Architecture Groups (MDAs) and their Sequence Alignments

A domain is often part of a larger protein containing other domains that may be contributing to the protein's overall function, thus alignments of the entire protein sequence are also useful. We group together domains within a superfamily sharing the same domain partners and multi-domain architecture (MDA). For each superfamily in the FunTree dataset, protein sequences having the same MDA are aligned. CATH-Gene3D defines the MDA of each protein by initially scanning the sequence against hidden Markov models built from CATH domains. Any unassigned sequence regions large enough to constitute a domain are checked against Pfam and if a non-overlapping Pfam domain is found then it is included in the MDA. Sequences with the same multi-domain architectures are clustered using ArchSchema [26]. The sequences of each cluster are then aligned using MAFFT [43], and the alignment used to perform the phylogenetic analysis.

### Phylogenetic Analysis

Some superfamilies can have an extremely large number (tens of thousands) of associated sequences. This can lead to problems in both generating the alignments and calculating the phylogenetic trees, so the sequences are first filtered to reduce redundancy and numbers. If a family contains more than a few hundred sequences, the sequences are filtered by taxonomic lineage and uniqueness of function, retaining only unique representatives of each. The alignments from the SSGs and MDAs are used to generate a phylogenetic tree built with TreeBest (as described in the methods for compiling the TreeFam database [44]). As this method incorporates species phylogenies to building gene trees, a species tree is generated using the NCBI taxonomic [23] definition of species relationships for those species found in the SSG/MDA.

Sequence, structural and functional data is collected from public repositories. Comparisons of metabolites are undertaken and presented with the phylogenetic tree. As it is not always clear the contribution of individual domains in a MDA, a search is undertaken to remove sequences with MDAs that have ambiguity about the contribution of the superfamily domain to the novel function (see Text S1).

## Supporting Information

Figure S1 **The FunTree Phylogenetic Tree of Phosphatidylinositol-Phosphodiesterase Structurally Similar Group.** Each leaf is annotated with identifiers for UnioprotKB and (if the sequence also has a structure) PDB, CATH and MACiE references. In addition, the E.C. number is shown along with the clustering of metabolites as coloured boxes where the colour of the box indicates the relative similarity using a rainbow-colouring scheme. At the end of each leaf the multiple domain architecture is also show as coloured bars, with the phosphatidylinositol-phosphodiesterase defining domain identified in red. Three groupings can be detected in the tree: the first (G1) is the phosphodiesterases involved in glycerol metabolism and the mammalian phosphatidylinositol phosphodiesterases. The second group (G2) out-lying to G1 consists of just the phosphatidylinositol phosphodiesterases from bacteria. The third group (G3) out-lying to the rest of the phylogeny is the phosphodiesterases acting on 2-lysophosphatidylcholine in the generation of spider venom. Two sequences indicated by * are included in the tree which have structural data classified by CATH but are not included in the ArchSchema graph in Figure 1, as ArchSchema presents sequences found in the reviewed section of UniProtKB. FunTree includes sequences with structures with known function but not part of the reviewed section of UniProtKB as exclusion of such data would lead to a paucity of structural data (see ‘Generating Structurally Similar Groups’ section in Materials and Methods).
(EPS)

Figure S2 **Inositol-1,4,5-trisphosphate Bound to a Structure of a Mammalian Phosphoinositide-specific phospholipase C.** A LigPlot diagram of the residues interacting with the bound inositol-1,4,5-trisphosphate cognate ligand of phosphoinositide-specific phospholipase c-delta1 from rat (PDB ID 1djx). The residues directly hydrogen bonding (indicated by a dashed green line) with the ligand as well, as the ligand itself, are shown as ball and stick. Below are shown the residues identified in the plot mapped on the multi-domain architecture. It can be seen that the second domain does not participate in any interaction with the liagnd or active site and does not directly affect the enzymatic function.
(EPS)

Figure S3 **The Different Mechanisms Undertaken by Phosphatidylinositol-Phosphodiesterase Superfamily.** The first split is between the case where a histidine acts as a nucleophile (EC 3.1.4.41, Spider) and the histidines acting as general acid bases with a cyclic-phospho intermediate. There is a further split in the general acid/base class where, in eukaryotes, a metal ion holds the intermediate firmly in the active site, allowing hydrolysis to occur within the enzyme whereas, in the bacterial case, the metal is no longer present and so the intermediate diffuses from the active site as the cyclic-phospho species, and undergoes hydrolysis outside of the enzyme and thus is classified as a lyases (EC 4) rather than a hydrolase (EC 3).
(EPS)

Figure S4 **Changes in Functional Residues in the Phosphatidylinositol-Phosphodiesterase Superfamily.** The alignment of two representative structures, one from each of group 1 and group 3, using the structure based multiple sequence alignment used to generate the SSG phylogenetic tree of the phosphatidylinositol-phosphodiesterase superfamily. Residue conservation is highlighted in blue, with the graduation of the colour representing the level of conservation, as measured by ScoreCons. Catalytic residues are highlighted in red.
(EPS)

Figure S5 **Changes in Functions within Two Example Superfamilies.** Heatmaps showing the cumulative changes in phosphatidylinositol-phosphodiesterase (**A**) and Ntn-type amide hydrolase (**B**) superfamilies, where a change is observed based on the differences in E.C. annotations at the class level in a superfamily. The colour intensity indicates the number of times a change in E.C. class occurs. The top right half of the matrix shows the total counts of changes in E.C. class, while the lower right half shows the counts obtained by using the phylogenetic tree to infer the order in which changes have occurred. Along the matrix diagonal the number of changes occurring at the subclass (2^nd^ level) of the E.C. classification within that class are shown, with the number of changes occurring at the 4^th^ level of the E.C. number shown in brackets.
(EPS)

Figure S6 **Structurally Similar Groups and Structural Diversity for the Ntn-Type Hydrolasing Superfamily.** A schematic dendrogram (top left) showing the relative similarity based on SAP scores between each of the CATH representative domains (CATH domain identifiers are given at the leaves). Below, the representatives superimposed based on the cores of the domains. The representative from each SSG is shown on the right.
(EPS)

Figure S7 **The Common Mechanism of the Ntn-Type Amide Hydrolasing Domain.** In the majority of cases the N-terminal nucleophile is cysteine (as shown here), however in the protease enzymes it is threonine and, in the case of glutaryl-7-aminocephalosporanic-acid acylase (EC 3.5.1.93) is it serine. The variability of the reactions seen in this superfamily come entirely from the transport of the ammonia product (of the glutamine hydrolysis reaction) to a second domain, which then utilises the ammonia in a second reaction. In the case of the hydrolase enzymes in this sub-superfamily, there are no second catalytic domains associated with the mechanism, and so we see limited variability in the reactions.
(EPS)

Figure S8 **Contextualising Domain Partners with the Ntn-Type Hydrolasing Domain.** Relationships between the Ntn-type hydrolasing defining domain and its various domain partners in the context of the extended multi-subunit molecular machines they form. Representatives of each of the MDAs found in the 3 SSGs are shown.
(EPS)

Figure S9 **The Effect of Multi-Domain Architecture on Function.** For each superfamily, the fraction of its E.C.s found in a single domain is shown in green and those found in combination with other domains (MDA) in blue. Clearly novel function can be brought to any protein by adding another domain or set of domains (determined by combinatorial searching of all linear domain combinations to determine if the function is assigned to that domain combination). The proportion of functions that results from this domain addition is not coloured.
(EPS)

Figure S10 **Determining Contribution of Domains in MDA to Function.** For a superfamily domain (domain C), the functions for the multi-domain architectures that contain that domain are collected. To determine if the function is associated solely with the MDA or if the function exists without the context of domain C, each of the possible linear combinations of other domains in the MDA are analysed for functional annotations. If the novel function, in this example F4, is found in a different MDA context then there is ambiguity about the contribution of domain C to the function.
(EPS)

Figure S11 **Changes in Function within 276 Superfamilies Based on Phylogenetic Trees.** **A.** A heatmap showing the cumulative changes in all superfamilies where a change is observed based on the differences in E.C. annotations at the class level in a superfamily using the phylogenetic tree to infer the order in which changes have occurred. The colour intensity indicates the number of times a change in E.C. class occurs. As exchanges can occur more than once in a tree the number of observations are elevated compared to a simple all-by-all comparison (shown as the superscript number). For example, in the exchanges occurring within the E.C.1 class, 651 more observations are made than the 646 made in the all-by-all matrix. In addition, exchanges that occur between SSGs and therefore phylogenetic trees, are not counted (shown as a negative subscript number). Thus for the exchanges occurring within the E.C. 1 class, 85 exchanges are not accounted for. This results in 1,212 observations being made (646+651−85 = 1,212). **B.** A heatmap showing the cumulative changes in all superfamilies of enzymes with a single domain where a change is observed based on the differences in E.C. annotations at the class level in a superfamily. The colour intensity indicates the number of times a change in E.C. class occurs. The top right half of the matrix shows the percentage of changes (with total counts in brackets) of changes in E.C. class observed across all 276 superfamilies, while the lower right half shows the percentage of changes expected based on a random simulation of E.C. changes. Along the matrix diagonal the number of changes occurring within the E.C. class at the 4^th^ level of the E.C. number. **C.** The same exchanges as described for A but concentrating on the interchanges between classes. The top right of the matrix shows the observed percentage of changes, with actual count totals in brackets, and a random model of expected interchanges in the lower left of the matrix.
(EPS)

Figure S12 **General Reactions for Each E.C. Class.** Generalised reactions for each of the main classes in the E.C. classification and the average number of steps in each class (as catalogued by MACiE).
(EPS)

Text S1 **Supporting Material and Methods and Results sections.** The Methods section outlines the data collection of sequence, structure and functional data. It also describes the comparison of small molecule metabolites and determination of a domain's contribution to function in a multi-domain architecture. The Results section highlights the details of the changes in function within the first clade of the phosphatidylinositol-phosphodiesterases superfamily as well as details of the reaction mechanisms of the Ntn-type amide hydrolasing superfamiliy.
(DOC)
