# Supplementary material for: Exploring the Evolution of Novel Enzyme Functions within Structurally Defined Protein Superfamilies
Source: PLoS Comput Biol. 2012 Mar 1;8(3):e1002403. doi: 10.1371/journal.pcbi.1002403 (PMC3291543; doi:10.1371/journal.pcbi.1002403)
Supplement: Text S1 — Supporting Material and Methods and Results sections. The Methods section outlines the data collection of sequence, structure and functional data. It also describes the comparison of small molecule metabolites and determination of a domain's contribution to function in a multi-domain architecture. The Results section highlights the details of the changes in function within the first clade of the phosphatidylinositol-phosphodiesterases superfamily as well as details of the reaction mechanisms of the Ntn-type amide hydrolasing superfamiliy. (DOC) [file pcbi.1002403.s013.doc]

**TEXT S1:**

**Exploring the Evolution of Novel Enzyme Functions within Structurally Defined Protein Superfamilies**

**Results**

**Phosphatidylinositol-phosphodiesterases Superfamily**

**Detailed Summary Of The First Clade Of The Structurally Similar Group Of The Phosphatidylinositol-phosphodiesterases Superfamily**

The phylogentic tree of the structurally similar groups of the phosphatidylinositol-phosphodiesterases superfamily has three main clades. The first contains the phosphodiesterases involved in glycerol metabolism and the mammalian phosphatidylinositol-phosphodiesterases. Within this clade there are five functional sub-groupings, where the change in function is at the 4^th^ level of the E.C. number. The first subgroup contains a generalised glycerophosphodiester (E.C. 3.1.4.46) acting on the ester bond to generate a glycerol phosphate and an alcohol. Another generalised functional group (E.C. 3.1.4.1) performs a hydrolysis of the 3’ termini of oligonucleotides, though the functional assignment has been made using predictive methods and does not have any experimental data to support its validity. The third and fourth have inositol as a specific R group to the glycerophosphodiestester (3.1.4.43 / 3.1.4.44) and, depending on which ester bond is broken, generate either inositol and glycerol phosphate or inositol phosphate and glycerol. The fifth functional sub-group uses a glycerophosphodiestester with an inositol with 2 phosphates attached (E.C. 3.1.4.11) to generate D-myo-inositol-1,4,5-trisphosphate and sn-1,2-diacylglycerol. These products are secondary messengers in eukaryotic signal transduction cascades which release calcium stores and control protein phosphorylation by activation of protein kinase C[1]. The generalised functions (E.C. 3.1.4.1 and 3.1.4.46) as well as those that use inositol glycerophosphodiestester (without the additional phosphates on the inositol ring) as the substrate are found in both bacteria and eukaryotes, while the reactions that utilize the bi-phosphate inositol are only found in eukaryotes.

**Ntn-type Amide Hydrolasing Superfamily**

**Summary Of Reaction Mechanisms**

The Ntn-type amide hydrolasing domain performs its function in a mostly conserved manner, by the nucleophilic addition of an N-terminal residue to the carbonyl carbon portion of the amide bond. Except for the hydrolase cases (E.C. 3), in which the catalytic nucleophile is either a threonine residue (in the case of the proteasome) or a serine, these enzymes all utilise the N-terminal cysteine, which is activated by its own N-terminal amine. Their substrate is almost exclusively glutamine (except for the protease enzymes in which case it is a peptide). These enzymes then release ammonia and the substrate is hydrolysed off the enzyme. Again, the hydrolase enzymes differ in that the hydrolysis of the enzyme’s product is the final step in the reaction. In the other cases, the ammonia is then transported through an intramolecular channel to the second domain [2], where it can be used. It is in this second domain that the diversity of function really occurs. In the case of the ligases, a synthase domain catalyses the formation of a bond between two heavy atoms with the concomitant hydrolysis of ATP, thus we see the formation of asparagine from aspartate and ammonia from glutamine hydrolysis.The second domain often acts without the glutaminase activity, as can be seen from the example of E.C. 6.3.3.4 in which the glutaminase domain has lost its functional N-terminal residue. However, the synthase domain remains active, also in the case of E.C. 2.6.1.16, which is known to use water when the ammonia from the glutaminase activity is unavailable.

**Materials and Methods**

**Data Collection And Processing**

**Sequence, Structure and Functional Data Collection**

For each of the known domain structures in the SSGs the following identifiers are collected; the Protein Data Bank (PDB) identifier; the corresponding UniProtKB identifier; the CATH domain identifier and the functional identifier as defined by the Enzyme Commission (E.C.) number. If UniProtKB does not annotate the E.C. number, then the E.C. number as annotated in the PDB deposition (if present) is used. In addition, structural information including secondary structure and catalytic residues from PDBsum [3] and the Catalytic Site Atlas [4], respectively, are collected. Where a sequence does not have a known structure, only the functional information from UniProtKB is recorded.

**Small Molecule Comparisons**

The E.C. numbers are used to define the reactions being undertaken using the IUBMB enzyme nomenclature, which classifies enzymes by the reactions they catalyse. The small molecules defined in the reaction are collected and compared to each other across each superfamily and protein or domain family using the Small Molecule Subgraph Detector (SMSD) tool kit [5]. The resulting comparison matrix is clustered using PVCLUST [6] implemented in the R statistical package, and similarity trees are rendered using the software developed in-house for FunTree.

**Analysis Of Domain Contribution To Function In MDAs**

A novel function maybe performed by an enzyme associated with a domain superfamily where the function is connected with one or more domains, which do not possess the superfamily domain. To analyse the contribution of this phenomenon a combinatorial approach was taken. Each of the possible sequential domain combinations within a MDA is searched using ArchSchema to determine if the function is associated with that domain combination. If not then the function is uniquely associated with the MDA; otherwise there is ambiguity about the contribution of the superfamily domain to the novel function (a summary is shown Figure S10).

**REFERENCES**

1. Essen LO, Perisic O, Katan M, Wu Y, Roberts MF, et al. (1997) Structural mapping of the catalytic mechanism for a mammalian phosphoinositide-specific phospholipase C. Biochemistry 36: 1704-1718.

2. Teplyakov A, Obmolova G, Badet B, Badet-Denisot MA (2001) Channeling of ammonia in glucosamine-6-phosphate synthase. J Mol Biol 313: 1093-1102.

3. Laskowski RA (2009) PDBsum new things. Nucleic Acids Res 37: D355-359.

4. Porter CT, Bartlett GJ, Thornton JM (2004) The Catalytic Site Atlas: a resource of catalytic sites and residues identified in enzymes using structural data. Nucleic Acids Res 32: D129-133.

5. Rahman S, Bashton M, Holliday G, Schrader R, Thornton J (2009) Small Molecule Subgraph Detector (SMSD) toolkit. Journal of Cheminformatics 1: 12.

6. Suzuki R, Shimodaira H (2006) Pvclust: an R package for assessing the uncertainty in hierarchical clustering. Bioinformatics 22: 1540-1542.
